# Integrated Genomics Identifies Five Medulloblastoma Subtypes with Distinct Genetic Profiles, Pathway Signatures and Clinicopathological Features

**DOI:** 10.1371/journal.pone.0003088

**Published:** 2008-08-28

**Authors:** Marcel Kool, Jan Koster, Jens Bunt, Nancy E. Hasselt, Arjan Lakeman, Peter van Sluis, Dirk Troost, Netteke Schouten-van Meeteren, Huib N. Caron, Jacqueline Cloos, Alan Mršić, Bauke Ylstra, Wieslawa Grajkowska, Wolfgang Hartmann, Torsten Pietsch, David Ellison, Steven C. Clifford, Rogier Versteeg

**Affiliations:** 1 Department of Human Genetics, Academic Medical Center, Amsterdam, the Netherlands; 2 Department of Neuropathology, Academic Medical Center, Amsterdam, the Netherlands; 3 Department of Pediatric Oncology, Academic Medical Center, Amsterdam, the Netherlands; 4 Department of Pediatric Oncology/Hematology, VU University Medical Center, Amsterdam, the Netherlands; 5 Department of Pathology, VU University Medical Center, Amsterdam, the Netherlands; 6 Department of Pathology, Children's Memorial Health Institute, Warsaw, Poland; 7 Department of Neuropathology, University of Bonn, Bonn, Germany; 8 Department of Pathology, St Jude Children's Research Hospital, Memphis, Tennessee, United States of America; 9 Northern Institute for Cancer Research, University of Newcastle, Newcastle-upon-Tyne, United Kingdom; University of the Western Cape, South Africa

## Abstract

**Background:**

Medulloblastoma is the most common malignant brain tumor in children. Despite recent improvements in cure rates, prediction of disease outcome remains a major challenge and survivors suffer from serious therapy-related side-effects. Recent data showed that patients with WNT-activated tumors have a favorable prognosis, suggesting that these patients could be treated less intensively, thereby reducing the side-effects. This illustrates the potential benefits of a robust classification of medulloblastoma patients and a detailed knowledge of associated biological mechanisms.

**Methods and Findings:**

To get a better insight into the molecular biology of medulloblastoma we established mRNA expression profiles of 62 medulloblastomas and analyzed 52 of them also by comparative genomic hybridization (CGH) arrays. Five molecular subtypes were identified, characterized by WNT signaling (A; 9 cases), SHH signaling (B; 15 cases), expression of neuronal differentiation genes (C and D; 16 and 11 cases, respectively) or photoreceptor genes (D and E; both 11 cases). Mutations in β-catenin were identified in all 9 type A tumors, but not in any other tumor. PTCH1 mutations were exclusively identified in type B tumors. CGH analysis identified several fully or partly subtype-specific chromosomal aberrations. Monosomy of chromosome 6 occurred only in type A tumors, loss of 9q mostly occurred in type B tumors, whereas chromosome 17 aberrations, most common in medulloblastoma, were strongly associated with type C or D tumors. Loss of the inactivated X-chromosome was highly specific for female cases of type C, D and E tumors. Gene expression levels faithfully reflected the chromosomal copy number changes. Clinicopathological features significantly different between the 5 subtypes included metastatic disease and age at diagnosis and histology. Metastatic disease at diagnosis was significantly associated with subtypes C and D and most strongly with subtype E. Patients below 3 yrs of age had type B, D, or E tumors. Type B included most desmoplastic cases. We validated and confirmed the molecular subtypes and their associated clinicopathological features with expression data from a second independent series of 46 medulloblastomas.

**Conclusions:**

The new medulloblastoma classification presented in this study will greatly enhance the understanding of this heterogeneous disease. It will enable a better selection and evaluation of patients in clinical trials, and it will support the development of new molecular targeted therapies. Ultimately, our results may lead to more individualized therapies with improved cure rates and a better quality of life.

## Introduction

Medulloblastoma is a highly invasive primitive neuroectodermal tumor of the cerebellum. It is the most common malignant embryonal brain tumor in childhood with a peak incidence in the eighth year of life, but a significant proportion occurs in young adults and some even in elderly patients [1]. Current treatment includes surgical resection, radiotherapy and chemotherapy. Cure rates are improving, but survival is still poor and survivors suffer from serious long-term side effects of the therapy. Medulloblastoma patients are either classified as standard-risk patients (>3 years of age, non-metastatic disease and a totally or near-totally resected tumor) or otherwise as high-risk patients [1]. This risk stratification is unsatisfactory in predicting outcome, with under- or over-treatment as possible consequences. An improved disease sub-classification is therefore urgently required. In addition, a better insight into the molecular biology of medulloblastoma may lead to the development and application of novel therapies.

Cytogenetic studies and comparative genomic hybridization (CGH) studies have identified many chromosomal aberrations in medulloblastomas. Most frequent is isochromosome 17q, often in combination with loss of 17p, that is found in 30–50% of the cases [2]–[7]. These studies also identified high level amplifications of MYC family members in 5–15% of the cases, which is associated with a poor outcome [8]. Other proposed prognostic classifiers are expression of *MYC*, *TRKC*, *ERBB2*, nuclear localization of β-catenin or a large cell/anaplastic histology [9]–[18]. Mutations in Sonic Hedgehog (SHH) pathway genes (*PTCH1*, *SUFU*) are found in approximately 25% of medulloblastomas, and in WNT pathway genes (*β-catenin*, *APC*, *AXIN*) in approximately 15% of the cases [17], [19]–[23].

Early gene expression profiling studies, using serial analysis of gene expression, identified the homeobox gene *OTX2* as highly expressed and occasionally amplified in medulloblastoma [24]–[28]. The first microarray studies of medulloblastoma series used a supervised approach to identify differentially expressed genes after grouping for metastatic status, histology or survival [29]. Recent microarray analyses used an unsupervised approach to identify different molecular subtypes in medulloblastoma [30]. The most distinct subtype identified in this study was characterized by activation of the WNT signaling pathway, in most cases due to mutations in the β-catenin gene [30]. A second subtype, not completely separated from all other medulloblastomas in their cluster analyses, was found to be enriched in mutations in either *PTCH1* or *SUFU* and was characterized by activation of the SHH signaling pathway [30]. Other observed groups were not further characterized.

To further improve the molecular classification of medulloblastoma, we have studied a series of 62 medulloblastomas at the mRNA expression level by Affymetrix HG-U133 plus2.0 GeneChips and characterized genomic abnormalities in a sub-set of these (n = 52) by comparative genomic hybridization (CGH) analysis using 30 K oligonucleotide arrays. This integrated approach identified 5 molecular subtypes of medulloblastomas, each with a characteristic gene signature, specific clinicopathological features and some distinctive genetic aberrations.

## Materials and Methods

### Tissue samples and DNA/RNA preparation

In total 62 medulloblastoma samples were selected for Affymetrix gene expression profiling. The set included 60 primary tumors and 2 local relapses. Patient/tumor characteristics are presented in Table 1 for the whole series and in Table S1 for each sample separately. All samples were snap frozen in the institutional pathology departments immediately upon arrival. All samples were reviewed by experienced neuropathologists and examined for tumor content. Samples were excluded from analysis when less than 70% of the sample contained tumor cells. RNA and DNA was isolated using Trizol (Invitrogen, Carlsbad, CA). RNA purification was performed using the RNeasy mini kit (Qiagen, Germantown, USA). DNA and RNA quantity and quality was determined by spectrophotometry (Nanodrop, Wilmington, USA) and microfluidics-based electrophoresis (Agilent 2100 Bioanalyzer, Agilent, Palo Alto, USA).

**Table 1:** Patient/tumor characteristics of medulloblastoma series used for expression profiling in this study.

**Tumor samples**	
Primary tumors	60
Local relapses	2
**Sex**	
Male	41
Female	21
**Histology**	
Classic	46
Desmoplastic	13
LCA	1
ND	2
**Age**	
Median (range)	6.0 (1.5–35.3)
≤3 yr	14
>3 and ≤18 yr	41
>18 yr	6
ND	1
**Metastatic stage**	
M0	42
M1	7
>M2	9
ND	4

### Expression profiling

Four µg total RNA was used for hybridization of Affymetrix HG-U133 plus 2.0 Genechips according to the manufacturer's instructions (Affymetrix Inc. Santa Barbara, USA). Quality of the arrays was ensured by inspection of the beta-actin and GAPDH 5′-3′ ratio's as well as the percentage of present calls generated by the MAS5.0 algorithm (Affymetrix Inc. Santa Barbara, USA). For clustering and gene expression analyses data were normalized using the gcRMA algorithm in the Bioconductor package (http://www.bioconductor.org) of the R statistical environment (http://www.r-project.org/). Present call information from the MAS5.0 algorithm was added to the gcRMA gene expression measures. Expression data have been deposited in NCBI's Gene Expression Omnibus (GEO; http://www.ncbi.nlm.nih.gov/geo/) and are accessible through GEO Series accession number GSE10237. Expression profiles (.cel files) generated for the 46 medulloblastomas published by Thompson et al. [30] were kindly provided by Dr. Richard Gilbertson. They were also normalized using the gcRMA algorithm. Present call information came from the MAS5.0 algorithm.

### Expression data analysis

Affymetrix data showed for 43628 probe sets a present call in at least one of the tumors. They correspond to 17425 different genes. Unsupervised two-way hierarchical clustering and bootstrap analysis of tumor samples and expression data was performed with the TMEV program using Pearson correlation [31]. We used for clustering only one probe set per gene (the one with the highest average signal) and only probe sets with a present call in at least one of the tumors. Furthermore, we selected for clustering the smallest set of genes with the greatest variance in expression (variance filter in TMEV program) that showed the most robust cluster result with the highest support from the bootstrap analysis. Genes specifically expressed in each cluster of tumors found with cluster analysis were identified by comparing each cluster with all other tumors. For this we used the student t-test, corrected for multiple testing using the false discovery rate. Differentially expressed genes were categorized in GeneOntology groups using the DAVID tool (http://david.abcc.ncifcrf.gov/). Parametric analysis of geneset enrichment (PAGE) was performed for GeneOntology groups using the methods described by Kim and Volskey [32].

### Statistics

The CHI-square test was used to test the significance of clinical differences between subtypes. For differences in age at diagnosis we used the non-parametric Kruskal-Wallis test.

### CGH arrays

For 52 of the 62 medulloblastoma samples DNA was available for array CGH analysis. DNA quantity and quality was measured spectrophotometrically (Nanodrop, Wilmington, USA). Samples with an A260/230 ratio below 1.8 were not used for CGH analysis. For labeling 300 ng DNA was used, which was mixed with 300 ng labeled reference DNA of female origin. Labeled DNA was hybridized to Code-link glass slides which were spotted with 60mer oligonucleotides representing 28830 unique genes designed by Compugen (San Jose, CA, USA). Labeling, hybridization, and data analysis was all done according to the methods described by van den IJssel et al. [33]. The CGH array data were processed with the CGH-call algorithm in Bioconductor [34]. This algorithm generates amplified/neutral/deleted calls for all the probes on the CGH arrays. These calls were subsequently grouped on their cytoband position. The call which was most often encountered within a cytoband was recorded. All regions with gain were annotated as ‘+1’ (red) and regions with loss as ‘−1’ (green). Regions without aberrations were annotated as ‘0’ (white). We then performed a one-way hierarchical cluster analysis of these data for each molecular subtype separately. For this the TMEV program and Pearson correlation was used [31].

The CGH-call algorithm does not take chromosomes X and Y into account. Therefore, data for the X and Y chromosome were visualized in a different way. For all probes assigned to chromosome 1–22 the log2 ratio ch1/ch2 was recorded. Unassigned probes and probes assigned to the sex-chromosomes X and Y were not included. Subsequently, the median signal over all these probes was calculated. Data for all probes (including the probes from X and Y chromosome) were normalized by subtracting the median signal of probes from chromosome 1–22. Data for chromosome X and Y for each tumor were then visualized by plotting the average signal for chromosome X probes against the average signal for chromosome Y probes.

### Mutation analysis

*PTCH1*, *β-catenin*, *HRAS*, *KRAS*, and *NRAS* were sequenced using Big Dye Terminator v1.1 chemistry (Applied Biosystems, Applera France, Courtaboeuf, France). For *PTCH1* all 23 exons were sequenced. For *β-catenin* only exon 3 was sequenced. For *HRAS*, *KRAS* and *NRAS* exons containing codon 12, 13 and 61 were sequenced. Primer sequences can be found in Table S3. Sequencing was performed on an ABI 3730 capillary sequencer (Applied Biosystems, Applera France, Courtaboeuf, France). Forward and reverse sequences on electropherograms were analyzed using Codon Code aligner (Dedham, MA, USA).

## Results

### Expression profiling identifies 5 molecular subtypes in medulloblastoma

**Figure 1:**
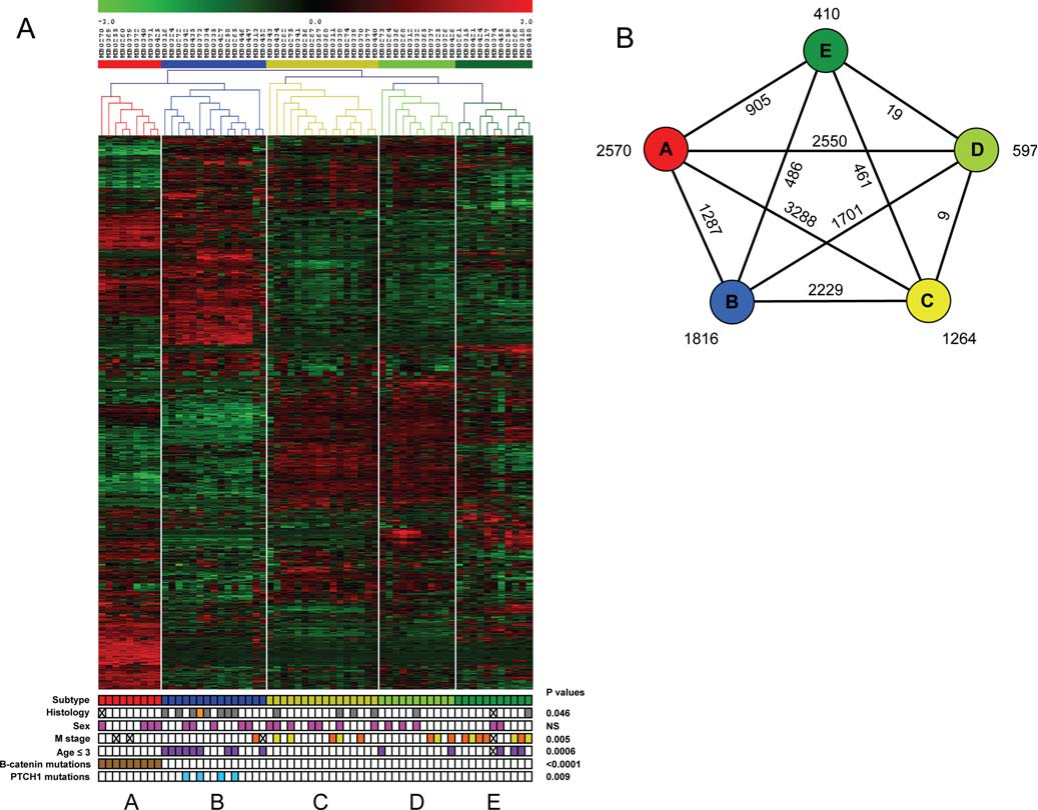
Identification of molecular subtypes in medulloblastoma. A. Unsupervised two-way hierarchical cluster analysis of 62 medulloblastoma samples and expression data of 1300 most differentially expressed genes identified 5 distinct clusters indicated as A, B, C, D, and E. Clinical annotations are at the bottom. Histology: grey = desmoplastic, orange = large cell/anaplastic, white = classic; sex: pink = female, white = male; Metastatic disease at diagnosis indicated with M stage: yellow = M1, orange = ≥M2, white = M0; Age: purple = age ≤3 yrs, white = age >3 yrs; *β-catenin* mutations: brown = mutations, white = wild type; *PTCH1* mutations: blue = mutations, white = wild type. A cross means not analyzed. B. Schematic pentagram showing the correlations between the 5 molecular subtypes of medulloblastoma. Numbers at the outside near each subtype indicate number of genes that are significantly differently expressed between that subtype and all other subtypes (P<0.001). Numbers at connecting lines indicate number of genes that are significantly differently expressed between medulloblastoma subtypes.

**Figure 2:**
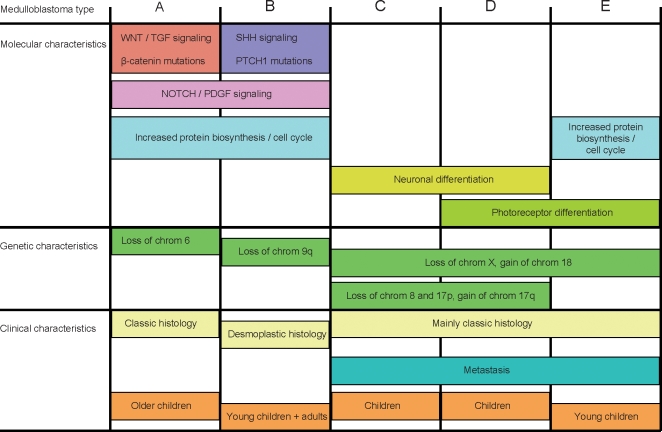
Schematic overview of the 5 molecular subtypes of medulloblastoma and their molecular, genetic, and clinical characteristics described in this paper.

We analyzed gene expression profiles of 62 medulloblastoma samples using Affymetrix HG-U133 plus 2.0 GeneChips. Patient and tumor characteristics are summarized in Table 1 and detailed in Table S1. Unsupervised two-way hierarchical cluster analysis identified 5 clusters, named A, B, C, D and E, consisting of 9, 15, 16, 11 and 11 samples, respectively (Figure 1A). Bootstrap analyses support differences between all 5 clusters, but clusters A and B stand out most clearly, while clusters C, D and E are more related to each other (Figure S1A and S1B). The specific characteristics of each molecular subtype are described below and are summarized in Figure 2.

### Cluster A is characterized by WNT and TGF-beta signaling

**Figure 3:**
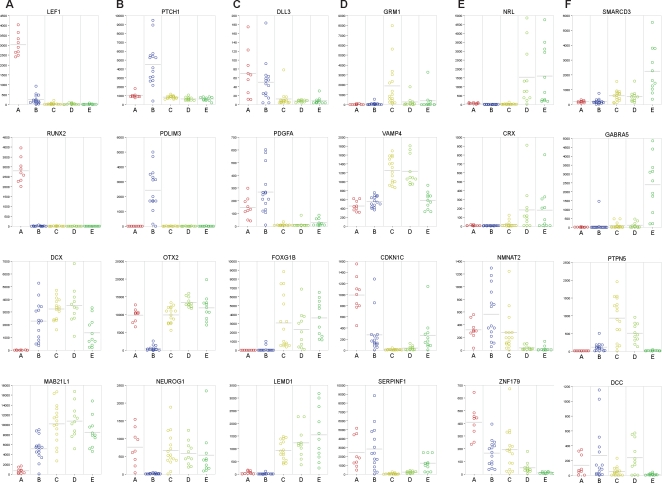
Examples of marker genes. For each medulloblastoma subtype A, B, C, D, and E, the expression data (vertical axes) are shown in each tumor (indicated with colored circles) for 2 markers that are specifically expressed or upregulated in that subtype, and 2 markers that are not expressed or only at very low levels in that subtype. A. Type A markers; B. Type B markers; C. In 2C markers are shown that are expressed either in subtype A and B together or in subtype C, D, and E together; D. Type C and/or CD markers; E. Type DE markers; F. Type E markers.

The smallest cluster of 9 tumors (cluster A, marked red in Figure 1) differed most strongly from all other tumors (Figure 1B). Many genes of the WNT pathway were overexpressed, including *AXIN2*, *LEF1*, *WIF1*, *KREMEN*, *DKK1*, *DKK2*, *DKK4*, *WNT11* and *WNT16* (Figure 3A and Table S2). Several of them are inhibitors of the WNT pathway and are upregulated in a negative feedback loop upon pathway activation. Also known targets of the WNT pathway, like *MYC*, *RUNX2*, *GAD1* and *SP5* were highly expressed in this group. The WNT pathway is known to be activated in a subset of medulloblastomas by *β-catenin* mutations [22], [35]–[37]. Indeed, mutations were found in all 9 tumors of cluster A, resulting in amino acid changes at positions 32, 33, 34, or 37 of the β-catenin protein. No such mutations were found in any of the other 53 samples (P<0.0001) (Figure 1A and Table S1). This cluster therefore corresponds to the medulloblastoma subtype with WNT pathway activation and β-catenin mutations identified by Thompson et al. [30] and Clifford et al. [38] (see also below). Further characteristics of this cluster were exclusive expression or overexpression of members of the TGFβ pathway, like *BMP4*, *BMP7*, *BAMBI*, *AMHR2*, *SMAD3*, *TGFBI* and *INHBA*.

### Cluster B is marked by SHH signaling and low levels of OTX2

Cluster B (15 samples, marked blue in Figure 1) is characterized by the overexpression of genes involved in SHH signaling such as *PTCH1*, *BOC*, and *GLI2* (Figure 2 and 3B and Table S2). *HHIP* and *GLI1* were also upregulated, but without reaching the statistical cut-off of P<0.001. Also known targets of the SHH signaling pathway such as *SFRP1* and *BCL2* were exclusively expressed or upregulated in this cluster. Activation of the SHH signaling pathway in medulloblastoma can occur by mutations in *PTCH1* [19], [39], [40]. Sequencing of all 23 exons of the *PTCH1* gene indeed identified frameshift mutations in 4 tumors of cluster B, but not in any of the remaining tumors (P = 0.009) (Figure 1A and Table S1 for mutation details). Interestingly, the oncogene *OTX2*, which is amplified in a low percentage of medulloblastomas, but highly expressed in about 75% of medulloblastomas [26]–[28], is not or hardly expressed in group B tumors (Figure 3B).

### Clusters A and B share increased expression of genes involved in protein biosynthesis, cell cycle, NOTCH and PDGF pathways

Although tumors from cluster A and B were very distinct from each other, they also share certain characteristics (Figure 2). Comparison of the combined 24 samples of the A and B cluster against all 38 other tumors identified 1484 genes significantly higher expressed in A and B (p<0.001) (Table S2). Major pathways recognized amongst them were the NOTCH and PDGF pathways. Genes like *NOTCH1*, *NOTCH2*, *DLL3*, *MNFG*, *MAML1* and *MAML2* were higher expressed in clusters A and B, although differences did not always reach the statistical cut-off (p-values between 0.05 and 0.001). For the PDGF signaling pathway we found increased expression of *PDGFA*, *PDGFC*, and *PDGFRA* in tumors of clusters A and B (Figure 3C). Also cell cycle genes (e.g. *CDK2* and *CDK2AP1*) and the protein biosynthesis machinery (e.g. ribosomal proteins) were upregulated (Figure 2 and 3C). Only cluster E also showed increased expression of cell cycle and protein biosynthesis genes (see below).

### C, D and E tumors

Tumors in clusters C, D and E are closely related. They fall apart in three clusters, based on expression of a series of neuronal differentiation genes in clusters C and D and expression of retinal differentiation genes in clusters D and E (see below and Figure 2). Comparison of all 38 tumors in clusters C, D, and E together with the 24 samples of the A and B cluster identified 1595 genes higher expressed in C, D, and E (Table S2). However, we did not detect signaling pathways or biological processes that were specifically activated in all tumors of the C, D and E clusters. Amongst the genes specifically expressed in all three clusters C, D and E were two transcription factors involved in brain development, *FOXG1B* and *EOMES*. Also *LEMD1*, a testis specific gene recently found to be overexpressed in colorectal cancer [41] and several genes involved in neuronal migration, like *UNC5D* and *EPHA8*, were specific for all tumors in clusters C, D and E (Figure 3C and Table S2).

### Cluster C and D are marked by increased expression of neuronal differentiation genes

Genes identified as overexpressed in cluster C were in most cases also overexpressed in cluster D, but not in cluster E (Table S2). Characteristic for these genes was that they were usually not completely silent in other clusters, but expressed at a lower level. Gene Ontology analysis showed that many overexpressed genes in C and D tumors were involved in neuronal differentiation (*NNAT*, *NEOROD2*, *RTN1*, *RTN4R*, *NEURL*, *NPAS2*, *DPYSL5*), cytoskeleton organization and biogenesis (*MPP3*, *MAST1*, *MAP2*) or vesicle mediated transport (*VAMP4*, *DNM1*, *DNM3*). Also members of the glutamate (*GRM1*, *GRM2*, *GRM8*) and GABA receptor family (*GABBR2*) were highly expressed in cluster C and D (Figure 3D and Table S2). Parametric analysis of geneset enrichment (PAGE) showed that several members of the MAPK signaling pathway, such as *KRAS*, *DUSP4*, *DUSP5*, *GRB2*, *FGF1*, *FGF9*, *FGF13*, and *FGF14* were expressed at higher levels in C and D tumors. As *RAS* mutations have been found at a low frequency in medulloblastomas [42], we sequenced the *KRAS*, *NRAS*, and *HRAS* genes for codon 12, 13 and 61. However, no such mutations were found in tumors from cluster C or D, nor in any other tumor, which is consistent with recent data from Gilbertson et al. [43]. Interestingly, the cell cycle inhibitor *CDKN1C* (*P57*) was not or hardly expressed in any tumor of cluster C and D, but is well-expressed in tumors of cluster E and most other tumors of clusters A and B (Figure 3D). PAGE analysis also showed that many other cell cycle related genes, such as *CCNB1*, *CCNB2*, *CDK6*, *CDKN2C*, were more weakly expressed in C and D tumors than in tumors of clusters A, B, and E (Figure 2). These analyses suggest that the cluster C and D tumors express a set of genes marking a certain neuronal differentiation stage or lineage and have relatively low cell cycle activity.

### Cluster D and E are marked by expression of retinal genes

When we compared the combined 22 tumors of cluster D and E with the 40 tumors of all other clusters, we found 197 genes significantly overexpressed in D and E (Table S2). A striking number of them were genes normally expressed in photoreceptor cells in the retina (Figure 2). Retina-specific transcription factors, such as *NRL*, *CRX* and *NR2E3* were expressed at moderate to high levels in the D and E clusters, but not in any other cluster (Figure 3E). Other genes encoding retinal antigens like *ROM1*, *SAG* (*S-antigen*), *AIPL1*, *RPGRIP1*, *TULP1*, and *PDE6H* were also exclusively expressed in cluster D and E.

Comparison of the E cluster with the D cluster showed E-specific expression of a series of genes like *SMARCD3* and *GABRA5* (Figure 3F). In addition, PAGE analysis showed that E tumors have an enriched expression of ribosomal protein genes and genes functioning in the cell cycle, amino-acyl tRNA biosynthesis and pyrimidine metabolism (Figure 2 and Figure S2).

### Array CGH analysis reveals subtype-specific DNA copy number aberrations

**Figure 4:**
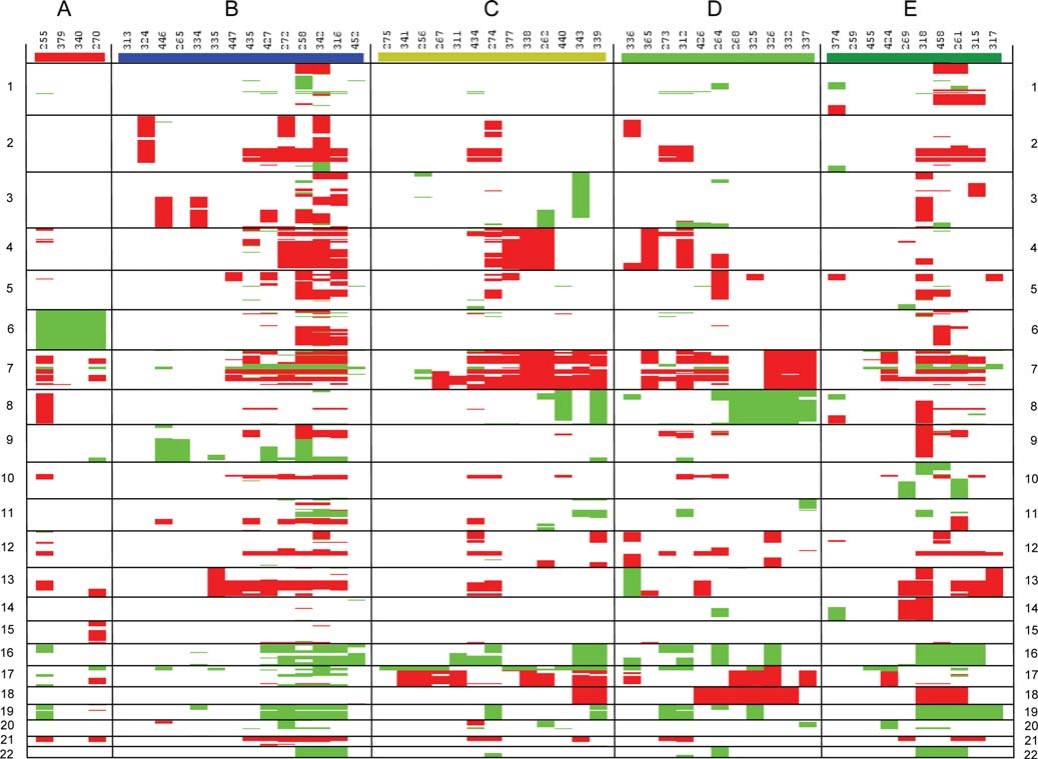
Cluster analysis of genetic aberrations in the different molecular subtypes identified with array CGH. Array probes were grouped together according to their position in distinct cytobands. Cytobands were then scored in each tumor as gained (+1, red), lost (−1, green), or unchanged (0, white), based on the array CGH data for all probes in a particular cytoband region. Subsequently, these cytoband data for gain, loss or no change were clustered using an unsupervised one-way hierarchical clustering. This was done for each molecular subtype separately.

For 52 of the 62 tumors sufficient high quality DNA was available for analysis by CGH arrays (type A: 4 samples, type B: 14 samples, type C: 13 samples, type D: 11 samples, type E: 10 samples). Many aberrations were detected with these arrays and the different subtypes show distinct patterns of gains and losses (Figure 4). All 4 analyzed tumors with a WNT signature (type A) lost a full copy of chromosome 6, but none of the other 48 tumors did (p<0.001). These data are consistent with data from Thompson et al. [30] and Clifford et al. [38]. Type B tumors (SHH type) showed frequent loss of 9q sequences (8/14 samples). In other clusters this occurred in only 3 other tumors (p = 0.001). Clusters C, D and E often showed gain of chromosome 18 (11/34 samples; p = 0.012), which was never found in clusters A and B. Gain of chromosome 17q, often in combination with deletion of chromosome 17p, was strongly associated with tumors in clusters C and D (13/24 samples; p = 0.002). Loss of chromosome 8 was exclusively found in tumors of clusters C and D (7/24 samples; p = 0.007). Gain of chromosomes 2, 4, 5, 7, 9, 12, 13, and 21 or parts of them, was also frequently found but they were not restricted to any particular subtype. This was also the case for the loss of regions on chromosomes 1, 11, 16, 19, and 22. Interestingly, in almost every subtype there seemed to be 2 groups of tumors: one group with very few aberrations and one group with many aberrations (Figure 4). Furthermore, we identified several high level amplifications of genes such as *MYCN*, *MYC*, or *MYCL1*. These amplifications occurred at low frequency and no statistically significant correlation was observed with any of the different subtypes.

**Figure 5:**
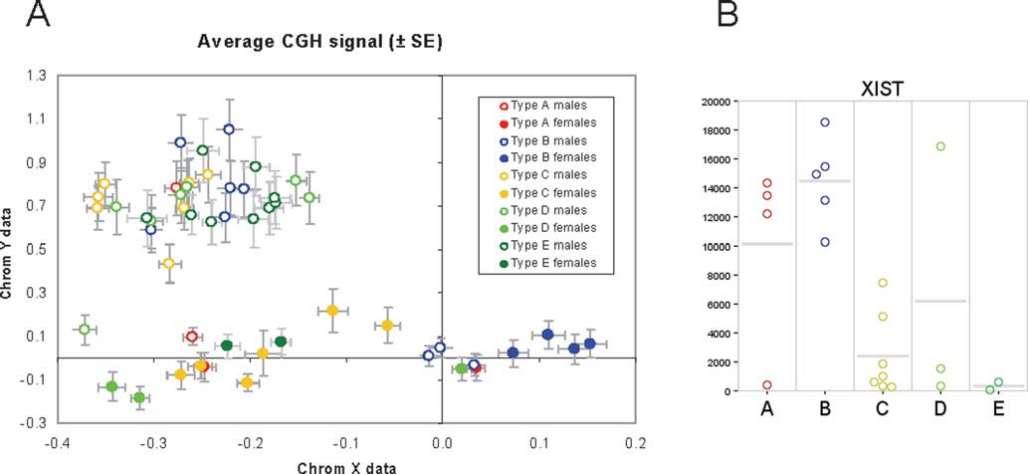
CGH analysis of sex chromosomes. A. One copy of chromosome X is lost in most female cases of clusters C, D, and E. The plot shows array CGH data for the X and Y chromosome in males and females of the different subtypes. The plotted data represent the average logratio for probes on chromosome X vs chromosome Y after normalization on the median of autosomes (see methods). The error-bars represent the standard error of the mean. Open circles represent male patients, closed circles are for female patients. Colors correspond to the colors for the different subtypes as indicated in Figure 1. B. *XIST* expression (vertical axis) is shown for 21 female cases (indicated with colored circles) in the MB62 series. 17 of them were analyzed by CGH arrays (Figure 5A). *XIST* expression is lost or strongly reduced in most type C, D, or E tumors, but not in type A or B tumors.

Loss of one of the X chromosomes (average logratio<−0.1) was found in 10/17 tumors of female patients (Figure 5A). In contrast, none of the tumors of male patients lost an X chromosome. *XIST*, which is specifically expressed from the inactivated X chromosome, was not or hardly expressed in 9/10 samples with loss of X, indicating that they lost the inactivated copy of the X chromosome (Figure 5B). The other sample showed reduced *XIST* levels. Figure 5B also shows *XIST* levels for 4 other female cases not analyzed by CGH. Strikingly, 9 out of 10 cases with almost complete loss of *XIST* expression were medulloblastomas from clusters C, D and E (p = 0.034). Only one of the analyzed female cases of cluster D retained 2 copies of the X chromosome and had high *XIST* expression. In the combined clusters A and B, only 1/9 tumors of female origin had lost the expression of *XIST* due to the loss of a copy of the X chromosome. Loss of the Y chromosome occurred in 5 out of 34 analyzed male samples and was not restricted to any subtype (Figure 5A).

### Chromosomal copy number changes correspond to strong expression differences

**Figure 6:**
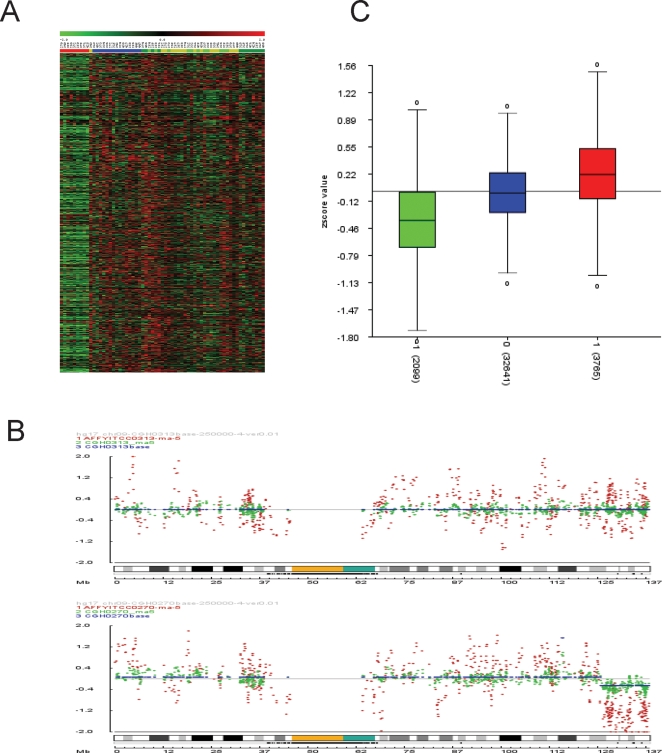
Genetic aberrations affect gene expression. A. Expression data suggest loss of chromosome 6 in all 9 tumors of cluster A. A one-way unsupervised hierarchical clustering of 62 medulloblastoma samples is shown for all expressed genes on chromosome 6. Colors on top correspond with the colors of the different subtypes as shown in Figure 1. Genes on the vertical axis are not clustered and are placed next to each other according to their position on the chromosome. B. Example of how DNA alterations affect gene expression. Chromosome 9 array CGH data (green) and expression data (Z-scores, red), both plotted on the Y-axis, are shown for two samples. MB0313 has no DNA alterations for chromosome 9, and MB0270 has lost the telomeric part of 9q. C. Overall analysis shows how genetic aberrations affect gene expression. Cytoband regions for all tumors analyzed by array CGH were marked as ‘−1’ for loss, ‘0’ for no change, and ‘+1’ for gain. Then the average expression (average Z-scores) of all expressed genes in these regions was calculated for each tumor and plotted on the Y-axis for each category. Results show that genes in regions with loss are on average expressed at lower levels (green box) and genes in regions with gain at higher levels (red box), compared to regions with no changes (blue box). Numbers at the X-axis between brackets represent the number of probe sets that were taken into account per category.

We finally performed an integrated analysis of expression data and DNA copy number data. A major question that we tried to answer was whether chromosomal gains and losses affect the overall expression levels of the affected chromosomal domains. First, we mapped all Affymetrix probe sets to the human genome sequence. We then performed for each chromosome separately an unsupervised one-way clustering of all tumor samples using the data for all expressed genes. Many regions came out as overall underexpressed or overexpressed and these regions showed a good correspondence with the CGH-analysis data. A striking example is chromosome 6 in the group A tumors: All 9 tumors showed about 50% lower expression of most genes of chromosome 6, compared to the other tumors (Figure 6A). As described above, deletion of one copy of entire chromosome 6 was confirmed by CGH in 4/4 analysed cases of group A tumors. Also, loss or gain of other chromosomal regions detected with array CGH, such as 8, 9q, 17p, 17q, or 18, clearly affect gene expression levels in these domains (Figure S3). Figure 6B shows an example how gene expression levels are strongly decreased in a tumor with 9q loss when compared to a tumor without 9q loss. Second, we compared all chromosomal regions with gain or loss directly with the gene expression levels in these regions. An overall analysis shows a good correlation for all chromosomal regions (P<0.0005) (Figure 6C). Average gene expression levels are higher in regions that are gained and lower in regions that are lost. This was true when all regions from all chromosomes were analyzed together, but also when the data were analayzed per chromosome (Figure S4). These data suggest that the distinct gene signatures are not only caused by mutations in signalling pathways, such as *β-catenin* or *PTCH1* mutations, but also by the subtype-specific chromosomal copy number aberrations.

### Metastatic tumors are predominantly found in clusters C, D, and E, but are not found in cluster A

The 5 molecular subtypes are marked by several clinical characteristics. The most striking finding was that presence of metastatic disease at diagnosis was strongly associated with clusters C, D, and E, as 15 of the 16 cases (94%, p = 0.005) with metastases were of these subtypes (Figure 1A). In cluster C the frequency was 31% (5/16 patients: 3 M1 and 2≥M2), in cluster D this was 27% (3/11 patients: 1 M1 and 2≥M2), while in cluster E this was 70% (7/10 patients: 3 M1 and 4≥M2). For 1 patient of cluster E we had no information on metastatic state. In contrast, none of the 7 samples of cluster A and only 1 of the 14 (7%; M2) samples of cluster B, for which data were available, had metastatic disease at diagnosis.

### Age at diagnosis and histology differ between the 5 subtypes

Medulloblastoma mainly affects children and is rare in adults. The median age at diagnosis for the whole series was 6.0 yrs, but varied significantly among the 5 subtypes (p = 0.006; Kruskal-Wallis test). Median age at diagnosis was 10.4 yrs (range 6–20) for cluster A, 3.0 yrs (range 1.5–35.3) for cluster B, 7.2 yrs (range 3.7–25.6) for cluster C, 5.9 yrs (range 3–16.6) for cluster D, and 3.8 yrs (range 2–15) for cluster E. Of the 14 patients below 3 yrs of age, 9 belonged to cluster B, 2 to cluster D and 3 to cluster E (p = 0.0006). Of the 4 patients of 25 yrs or older, 3 were found in cluster B and 1 in cluster C.

Also histology was significantly different between the 5 subtypes (p = 0.046). Most desmoplastic tumors were found in cluster B, some occurred also in other clusters, but never in cluster A (Figure 1A). In cluster A all tumors were of classic histology. There was only one tumor in our series with a large cell anaplastic histology, which was found in cluster B.

### Analysis of independent medulloblastoma dataset shows same molecular subtypes

Recently, Thompson et al. [30] published the results of an expression profiling study of 46 medulloblastomas. Their analysis also identified 5 clusters. One cluster showed WNT-pathway activation and 5 out of these 6 samples had β-catenin mutations. A second cluster (9 samples) was associated with SHH signaling and 4 samples had mutations in *PTCH1* and 1 in *SUFU*. A third cluster of 13 samples also included 4 tumors with an activated SHH signaling, one of them with a *PTCH1* mutation as well. However, the other clusters were not described in detail. The clustering of Thompson et al. did not identify clusters associated with metastatic disease at diagnosis, suggesting that the clusters identified in their study were not identical to the clusters identified in our study. The approach of Thompson et al. differed in two aspects from our study: 1) They used the Affymetrix HG-U133A GeneChip, while we used the more recent Affymetrix HG-U133 plus2.0 array, with twice as many probe sets; 2) Thompson et al. used expression values of all probe sets for clustering, while we used a set of most differentially expressed genes. We therefore decided to re-analyze the data of Thompson et al. using the approaches described in this paper.

**Figure 7:**
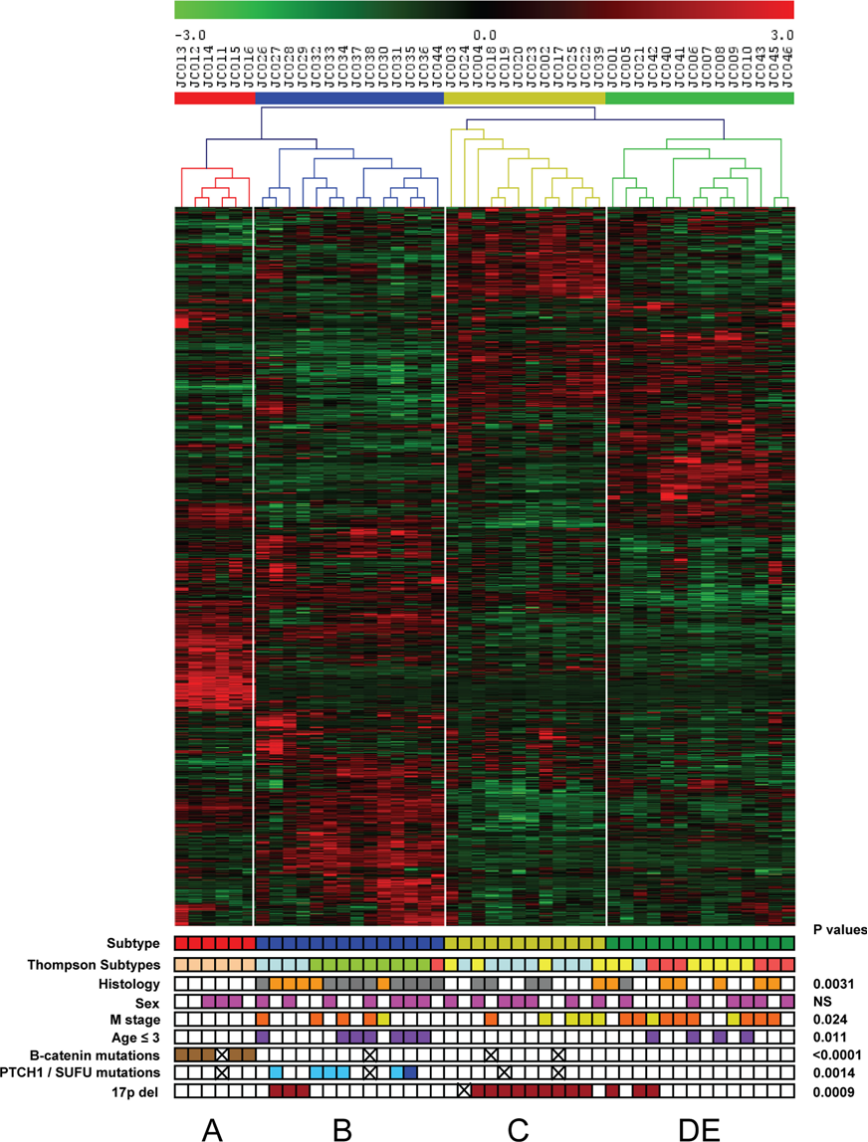
Unsupervised two-way hierarchical cluster analysis of 46 medulloblastoma samples and expression data of 1500 most differentially expressed genes from Thompson data series [30]. The clustering identified 4 distinct clusters indicated as A, B, C, and DE. Clusters D and E could not be separated in this series. Clinical annotations are at the bottom. Thompson subtypes as indicated in their paper: yellow = Thompson subtype A, light pink = Thompson subtype B, blue = Thompson subtype C, green = Thompson subtype D, dark pink = Thompson subtype E; Histology: grey = desmoplastic, orange = large cell/anaplastic, white = classic; sex: pink = female, white = male; M stage: yellow = M1, orange = ≥M2, white = M0; Age: purple = age ≤3 yrs, white = age >3 yrs; β-catenin mutations: brown = mutations, white = wild type; *PTCH1*/*SUFU* mutations: light blue = *PTCH1* mutations, dark blue = *SUFU* mutation, white = wild type; 17p deletions: dark red = yes, white = no. A cross means not analyzed.

We first clustered the Thompson data on basis of the 1500 genes/probe sets most differentially expressed in that series (note that these are not the same probe sets used in our study, as the HG133A chip has fewer probe sets). This analysis identified 4 distinct clusters, supported by bootstrap analysis (Figure 7 and Figure S5). T-tests performed to compare each cluster with the other 3 clusters showed that the first 3 clusters corresponded to our clusters A (6 samples), B (14 samples) and C (12 samples), while the remaining 14 tumors that grouped together corresponded to our D and E tumors (see Figure S6). Cluster A represented all WNT-signature samples. Interestingly, all samples for which Thompson et al. showed an SHH signaling profile, now clustered together in the B cluster. The data also suggested that the number of D type tumors is relatively low in the Thompson series. Expression of marker genes such as *GABRA5*, *SMARCD3*, *PLXNC1*, *DCC*, and *FGF9* (Figure 2 and Figure S6 and data not shown) strongly suggested that most tumors in the combined D/E cluster correspond to the E type. This is also illustrated by the strong association (p = 0.0009) of 17p deletions with tumors in cluster C (Figure 7), which is in line with our results that chromosome 17 aberrations are frequently found in type C and D tumors, but not or much less frequent in type A, B, or E tumors (Figure 4). These results show that similar subtypes are identified in both data sets and that the differences in clustering in the Thompson study and our study depend on the number of genes used for clustering and to some extend on differences in size and composition of both tumor series.

As a second comparison of the analyses, we also clustered the data from the Thompson series using the 1500 probe sets selected in our series. However, not all of these probes sets are present on the Affymetrix HG133A chip. Cluster analysis with expression data of the 897 probe sets that are present identified the same 4 clusters (A, B, C and D/E) in the Thompson dataset, which was supported by bootstrap analysis. Only one sample (JCO24) moved from cluster C to cluster D/E (Figure S7).

### Clinical characteristics in molecular subtypes are similar in both medulloblastoma datasets

Analysis of patient data for the 4 molecular subtypes we identified in the Thompson dataset confirmed our results for differences in metastasis, histology and age at diagnosis (Figure 7). Metastatic disease at diagnosis was observed with frequencies of 0% for cluster A, 36% for cluster B, 42% for cluster C and 71% for cluster D/E. Although the percentage of metastasis for cluster B is higher than in our series, these data fully confirm the identification of cluster C and D/E as tumors with the highest propensity for metastasis (p = 0.024), especially as most tumors in the D/E cluster probably represent the E type. The E type tumors are in both series strongly associated with metastatic disease at diagnosis. Tumors of cluster A clearly are devoid of metastases at diagnosis. As in our series, most desmoplastic tumors in the Thompson series were found in cluster B (9/14, p = 0.0031). Finally, also in the Thompson dataset, all patients below 3 years of age were found either in cluster B or D/E (p = 0.011) (Figure 7).

## Discussion

The clinical behavior of medulloblastoma cannot be fully understood without detailed knowledge of the intrinsic molecular biology. Here, we analyzed expression profiles in 62 tumors and DNA copy number alterations in 52 of them. We show that there are at least 5 distinct molecular subtypes (Figure 1 and 2). We validated and confirmed these data in an independent dataset of 46 medulloblastomas generated by Thompson et al. [30], with the exception that subtype D was underrepresented in their series and could not be separated from subtype E (Figure 7). Our data clearly demonstrate that medulloblastoma is a heterogeneous disease. Each subtype has its own characteristic gene-signature caused by different genetic aberrations and by mutations in pathway-specific genes as found for type A and B tumors. It is also apparent from our data that some characteristic signatures are present in more than one subtype. For instance, tumors in clusters C, D, and E are closely related and have partly overlapping expression patterns. Tumors in clusters C and D have in common that they overexpress many genes involved in neuronal differentiation and are low in cell cycle and protein biosynthesis related genes. Tumors in clusters D and E share a characteristic overexpression of many retinal antigens, but differ in cell cycle related genes (see the schematic overview in Figure 2). In contrast to the data of Thompson et al. [30] we show that not only tumors with WNT signaling (cluster A), but also tumors with SHH signaling (cluster B) form separate clusters. However, although distinct in many aspects, these 2 subtypes share the overexpression of genes involved in NOTCH and PDGF signaling. They also show an increased expression of genes involved in cell cycle and protein biosynthesis like the tumors in cluster E (Figure 2).

### Metastasis is associated with neuronal and photoreceptor specific gene-signatures

Analysis of the 5 clusters for associations with clinicopathological parameters showed a striking association with metastatic disease at diagnosis. Medulloblastoma has a high propensity for CNS spread and metastatic disease at diagnosis is a strong indication for poor outcome [44]. Consequently, patients with metastasis are treated with high-risk protocols. Our study showed that metastatic disease at diagnosis was strongly associated with subtypes C, D and especially E, while subtype A tumors never had metastatic disease at diagnosis. Our analysis is therefore in line with recent results of Ellison et al. [17] and Gaijar et al. [45] that medulloblastomas with activated WNT signaling, demonstrated by nuclear localization of β-catenin, have a favorable outcome and a very low frequency of metastatic disease at diagnosis. The genes and/or pathways that control the process of metastasis in medulloblastoma are largely unknown. Some studies suggested that increased PDGF signaling is associated with metastatic medulloblastomas [46], [47]. This is not supported by our results, as PDGF pathway members were mainly expressed in tumors of clusters A and B (Figure 3E). Comparison of the A and B clusters versus the C, D and E clusters identified differential expression of a series of genes with a potential role in neuronal migration and axon guidance (data not shown). Further analyses are required to value their role in metastasis.

### Desmoplastic tumors associated with young age and subtype B

The subtypes also differed in histology and median age at diagnosis. Subtype B tumors frequently showed a desmoplastic histology and were diagnosed in very young children or adults (Figure 2). Data from both medulloblastoma series together show that 16/25 tumors (64%) from infants were classified as type B and 12 of them had desmoplastic histology. The other 9 infants in both data series were all classified as either type D or E and none of them had desmoplastic histology (p = 0.0003). Recent reports suggest that desmoplastic tumors in infants have a very good prognosis [48]–[50]. We previously reported that medulloblastomas lacking expression of the *OTX2* oncogene are preferentially of desmoplastic histology and diagnosed in very young children and adults [28]. This is in good agreement with our current observation that *OTX2* is highly expressed in all medulloblastomas, except for cluster B tumors.

### Photoreceptor gene expression in medulloblastoma type D and E

A remarkable observation from our study is the expression of many photoreceptor-specific genes in type D and E tumors. Korf and colleagues already noticed in 1987 that a subset of medulloblastomas express *S-antigen* and *opsin*, both markers that are in a non-neoplastic state exclusively expressed in retinal photoreceptors and a class of pinealocytes which are derivatives of pineal photoreceptor cells [51], [52]. Several other studies confirmed these results [53]–[57], but the most recent paper on this topic is from 1995. Here, we show that not only *S-antigen* and *opsin* are specifically expressed in this subset of medulloblastomas, but many other photoreceptor-specific genes as well.

Retinal antigens are also expressed in retinoblastoma and tumors derived from pineal parenchyma (pineocytoma and pineoblastoma) [58]–[61]. This might suggest a common origin of these tumors and medulloblastomas with retinal antigens. Patients with hereditary retinoblastoma harboring a mutation in *RB1* are at increased risk of developing pineoblastoma, supratentorial PNET and there is one case report of a medulloblastoma in such a patient [62], [63]. Although *RB1* mutations have never been detected in other medulloblastomas [64], some experimental tumor models suggest a role for the RB pathway in this tumor. The combined loss of *p53* and *Rb1* in mouse external granular cells of the cerebellum resulted in medulloblastoma development [64]. Transgenic mice overexpressing SV40 large T-antigen develop, in addition to abnormal lens fiber differentiation and retinal dysplasia, also tumors resembling human pineoblastomas and/or medulloblastomas [65]. Most of these tumors express the photoreceptor markers *S-antigen* and *opsin* [51]. Large T-antigen can inhibit RB1, resulting in activation of E2F transcription factors and increased cell proliferation (reviewed by [66]). Recently, Olson et al. [67] showed that overexpression of *E2F1* in glial cells of transgenic mice also resulted in the induction of medulloblastomas and other PNETs. It will therefore be interesting to analyse a possible role of the RB1 pathway in medulloblastoma subtypes D and E with photoreceptor-specific gene expression.

### Chromosomal copy number changes strongly affect gene expression levels

We identified many recurrent chromosomal gains and losses in specific subtypes, such as the loss of one copy of chromosome 6 in all type A tumors or the loss of one copy of the X chromosome in most female cases in type C, D, or E tumors. Such defects suggest that tumor suppressor genes on these chromosomes play an important role in the tumorigenesis of these subtypes. However, our integrated expression and CGH study also showed that chromosomal copy number changes have a strong effect on the gene expression levels of almost all genes in the affected chromosomal areas. For example, this is striking for the expression of chromosome 6 genes in type A tumors, that have a reduction in expression equivalent to the reduction in copy number. Evidently, no compensatory mechanisms exist to increase the expression levels of most genes on the remaining copy. It is well established that gain or loss of a full chromosome has very strong consequences in embryogenesis. It either gives strong developmental defects, or is (as for most chromosomes) not compatible with life at all. The copy number changes that we observe in medulloblastoma may therefore also strongly contribute to pathogenesis. The consistent expression changes that we observed in the genes of the affected regions, may cause imbalances in the regulation of many pathways. Our integrated expression and copy number analysis provide data to further analyse the consequences of copy number changes for pathway activation or inactivation in medulloblastoma. In addition, the data might be applied to identify potential therapeutic target genes, whose role in tumorigenesis can be investigated with functional assays.

### Conclusions

Our study provides an integral overview of genetic aberrations and molecular pathway activation in medulloblastoma. Analysis of the expression profiles identifies 5 different types of medulloblastoma, each with characteristic activations of pathways and/or gene-signatures and each associated with specific genetic defects. These results raise the question whether these five subtypes all arise from the same cell lineage, but differ due to different genetic defects, or whether the five subtypes arise from distinct cell types and lineages. There is strong evidence that medulloblastomas with a SHH signature originate from progenitor cells of the external granular layer [68]. It is unknown whether other medulloblastomas also arise from these cells or whether they have another origin such as the ventricular zone [68]. Comparison of the profiles of the five medulloblastoma types with future expression profiles of progenitor cells involved in development of the cerebellum might answer this question. Regardless of the descent of the five subtypes, the specific genetic aberrations and the activation profiles of molecular pathways might help to improve clinical stratification of tumors and to guide selection of innovative therapies directed to specific gene products.

## Supporting Information

Figure S1 Bootstrap analyses. A. Bootstrap analysis of unsupervised two-way hierarchical cluster analysis (Pearson correlation) of MB62 data series using the 1300 most differentially expressed genes. Clusters A and B stand out most clearly, while clusters C, D, and E are more closely related. With 1300 genes bootstrap support is 97–100% that the 3 clusters A, B, and C are different from D and E, but the support for the difference between clusters D and E is only 21% with this number of genes. When we used 1500 genes bootstrap support for the difference between E and CD increased to 72% (see Figure S1B). B. Bootstrap analysis of unsupervised two-way hierarchical cluster analysis of MB62 data series using the 1500 most differentially expressed genes. With 1500 genes, clusters A and B still stand out most clearly and bootstrap support is 96–100% that clusters A and B are different from C, D, and E. However, in contrast to the results with 1300 genes (Figure S1A), bootstrap support for the difference between E and CD now increased to 72%. The support for the difference between C and D with these 1500 genes is 41%.
(0.80 MB PPT)

Figure S2 PAGE analysis for clusters D and E in MB62 dataseries shows higher levels of expression for cell cycle genes and other proliferation related genes in cluster E. Red dots in the graphs show the average expression for a gene in each cluster. The grey clouds around each dot indicate the standard deviation of expression.
(0.27 MB PPT)

Figure S3 Genetic aberrations suggested by expression data. A one-way unsupervised hierarchical clustering of 62 medulloblastoma samples is shown for each chromosome using all expressed genes on that particular chromosome. Colors on top correspond with the colors of the different subtypes as shown in Figure 1. The genes on each chromosome are not clustered, but are placed according to their position on the chromosome.
(3.27 MB PPT)

Figure S4 Genetic aberrations affect gene expression. Cytoband regions for all tumors analyzed by array CGH were marked as ‘−1’ for loss, ‘0’ for no change, and ‘+1’ for gain. Then the average expression (average Z-scores) of all expressed genes in these regions was calculated for each tumor and plotted for each category. Results show that genes in regions with loss are on average expressed at lower levels (green boxes) and genes in regions with gain at higher levels (red boxes), compared to regions with no changes (blue boxes). Numbers at the X-axis between brackets represent the number of probe sets that were taken into account per category. Data are shown for each chromosome apart. Chromosome numbers are shown on top.
(0.14 MB PPT)

Figure S5 Bootstrap analysis. Bootstrap analysis of unsupervised two-way hierarchical cluster analysis of MB46 data series of Thompson et al. [30] using the 1500 most differentially expressed genes shows that clusters A, B, C, and D/E are indeed distinct clusters. Bootstrap support is 88–100% that the 4 clusters are different from each other.
(0.43 MB PPT)

Figure S6 Expression data of same selected marker genes from Figure 3 in molecular subtypes of MB46 data series of Thompson et al. [30] show that the subtypes identified in the MB46 data series represent the same subtypes as identified in the MB62 data series. Expression data (vertical axes) are shown for each tumor (indicated with colored circles) for each medulloblastoma subtype. A. Type A markers; B. Type B markers; C. In 2C markers are shown that are expressed either in subtype A and B together or in subtype C, D, and E together; D. Type C and/or CD markers; E. Type DE markers; F. Type E markers. Expression of RUNX2, OTX2, LEMD1, and ZNF179 cannot be shown for the MB46 data series, since the HG-U133A GeneChip does not contain probe sets for these genes.
(0.44 MB PPT)

Figure S7 Cluster analysis. A. Unsupervised two-way hierarchical cluster analysis of 46 medulloblastomas from Thompson data series [30] using the 897 genes present at the HG-U133A GeneChip and present in the 1500 gene dataset selected as most differentially expressed in the MB62 data series. Results show that also with the genes selected from the MB62 data series 4 distinct clusters are identified in the MB46 data series containing the same samples as in Figure 7. Only one sample (JCO24) switched from cluster C to cluster D/E. B. Bootstrap analysis of unsupervised two-way hierarchical cluster analysis of MB46 data series [30] using the 897 genes present at the HG-U133A GeneChip and present in the 1500 gene dataset selected as most differentially expressed in the MB62 data series. The results show that also when these genes are used for clustering the 4 clusters are distinct clusters.
(0.33 MB PPT)

Table S1 Patient/tumor characteristics of medulloblastoma series used for expression profiling in this study.
(0.03 MB RTF)

Table S2 Gene-signatures for clusters A–E, and clusters AB vs CDE, CD vs ABE, and DE vs ABC.
(1.11 MB XLS)

Table S3 Primer sequences used for sequencing β-catenin, PTCH1, HRAS, NRAS, and KRAS.
(0.03 MB XLS)
