# Supplementary figures and images for: Integrated Genomics Identifies Five Medulloblastoma Subtypes with Distinct Genetic Profiles, Pathway Signatures and Clinicopathological Features
Source: PLoS One. 2008 Aug 28;3(8):e3088. doi: 10.1371/journal.pone.0003088 (PMC2518524; doi:10.1371/journal.pone.0003088)

## Slide 1
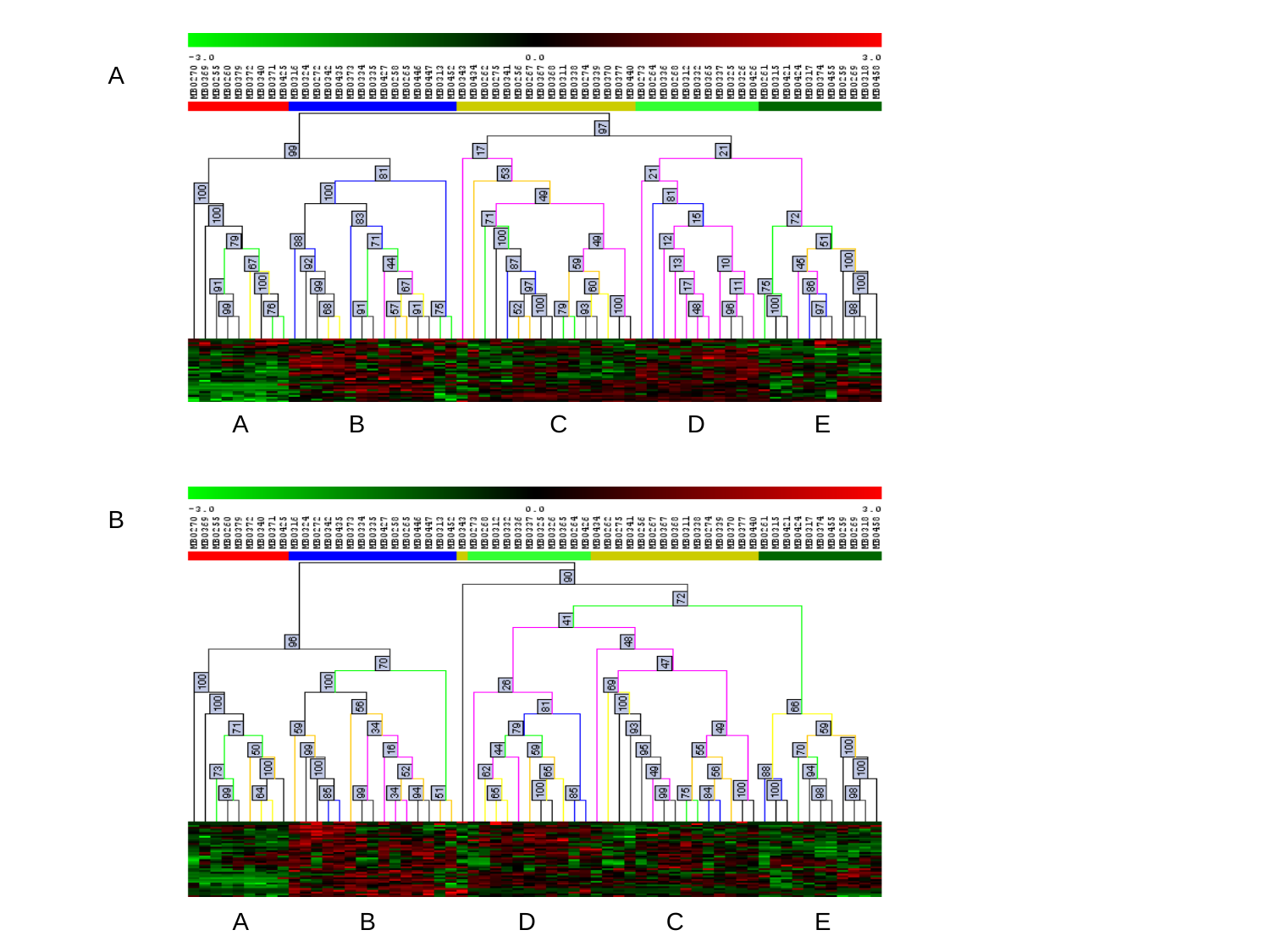

A
A
B
C
D
E
B
A
B
D
C
E

Supplement: Figure S1 — Bootstrap analyses. A. Bootstrap analysis of unsupervised two-way hierarchical cluster analysis (Pearson correlation) of MB62 data series using the 1300 most differentially expressed genes. Clusters A and B stand out most clearly, while clusters C, D, and E are more closely related. With 1300 genes bootstrap support is 97–100% that the 3 clusters A, B, and C are different from D and E, but the support for the difference between clusters D and E is only 21% with this number of genes. When we used 1500 genes bootstrap support for the difference between E and CD increased to 72% (see Figure S1B). B. Bootstrap analysis of unsupervised two-way hierarchical cluster analysis of MB62 data series using the 1500 most differentially expressed genes. With 1500 genes, clusters A and B still stand out most clearly and bootstrap support is 96–100% that clusters A and B are different from C, D, and E. However, in contrast to the results with 1300 genes (Figure S1A), bootstrap support for the difference between E and CD now increased to 72%. The support for the difference between C and D with these 1500 genes is 41%. (0.80 MB PPT) [file pone.0003088.s001.ppt]

## Slide 1
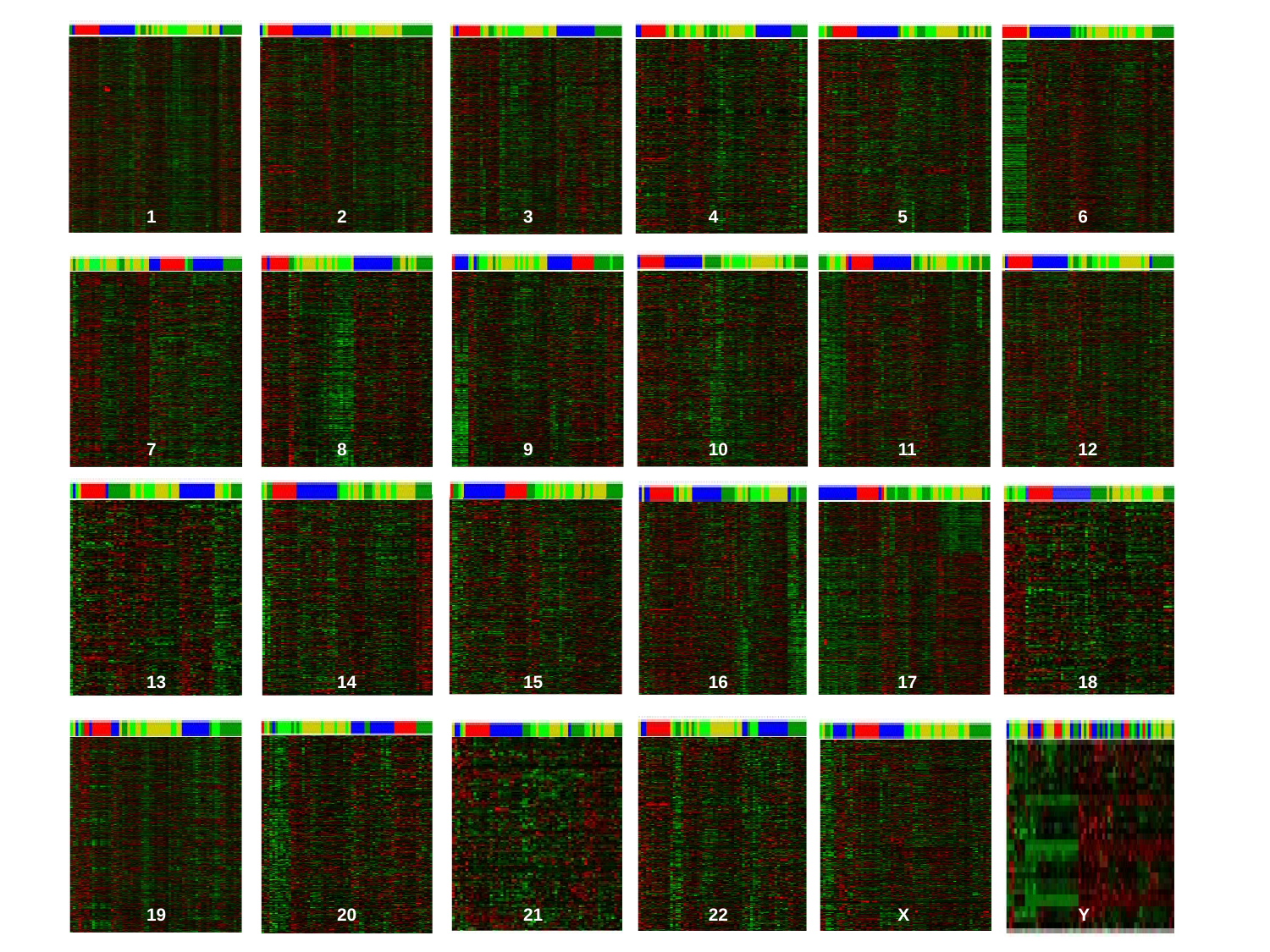

1
2
3
4
5
6
7
8
9
10
11
12
13
14
15
16
17
18
19
20
21
22
X
Y

Supplement: Figure S3 — Genetic aberrations suggested by expression data. A one-way unsupervised hierarchical clustering of 62 medulloblastoma samples is shown for each chromosome using all expressed genes on that particular chromosome. Colors on top correspond with the colors of the different subtypes as shown in Figure 1. The genes on each chromosome are not clustered, but are placed according to their position on the chromosome. (3.27 MB PPT) [file pone.0003088.s003.ppt]

## Slide 1
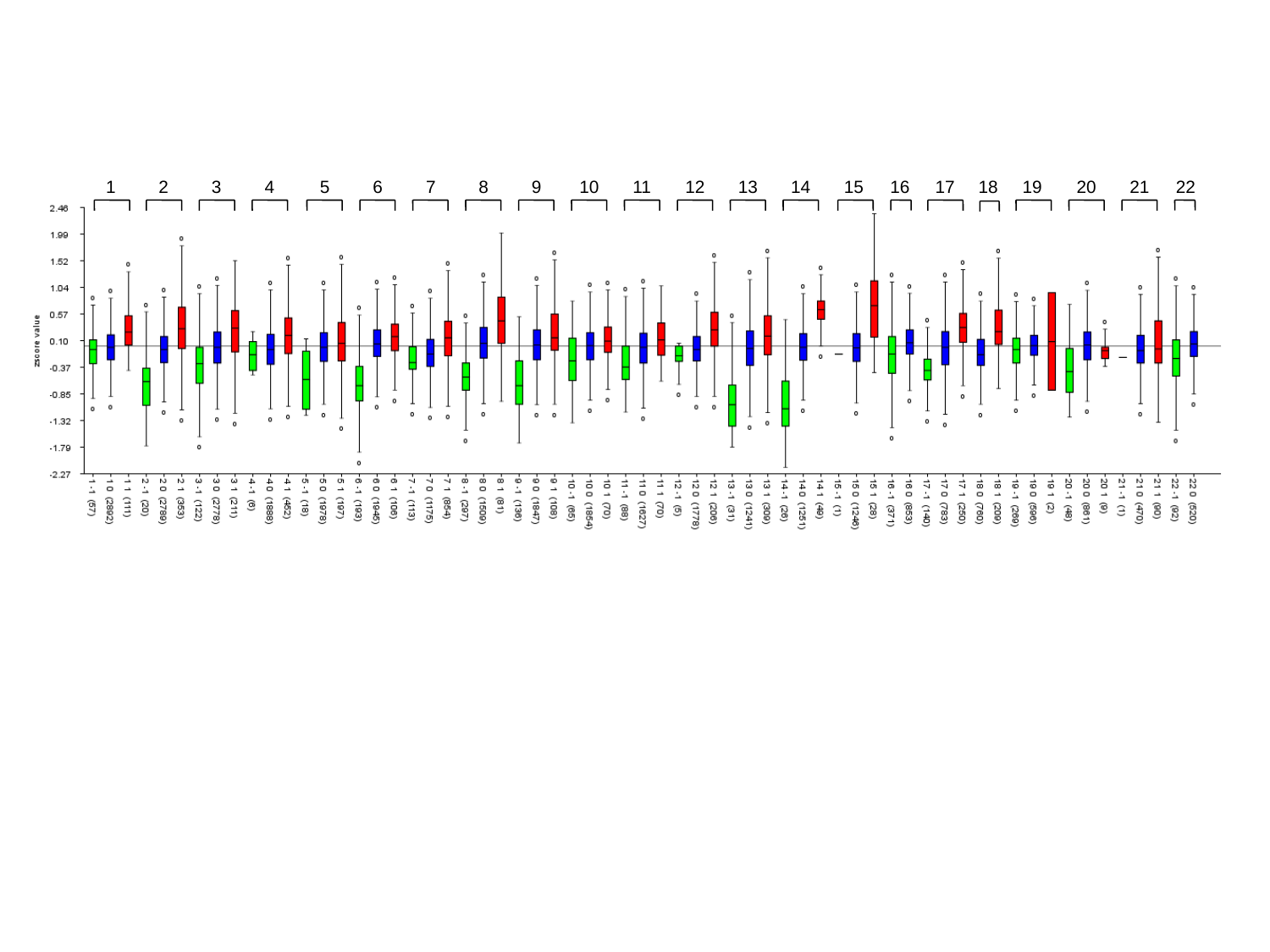

1
2
3
4
5
6
7
8
9
10
11
12
13
14
15
16
17
18
19
20
21
22

Supplement: Figure S4 — Genetic aberrations affect gene expression. Cytoband regions for all tumors analyzed by array CGH were marked as ‘−1’ for loss, ‘0’ for no change, and ‘+1’ for gain. Then the average expression (average Z-scores) of all expressed genes in these regions was calculated for each tumor and plotted for each category. Results show that genes in regions with loss are on average expressed at lower levels (green boxes) and genes in regions with gain at higher levels (red boxes), compared to regions with no changes (blue boxes). Numbers at the X-axis between brackets represent the number of probe sets that were taken into account per category. Data are shown for each chromosome apart. Chromosome numbers are shown on top. (0.14 MB PPT) [file pone.0003088.s004.ppt]

## Slide 1
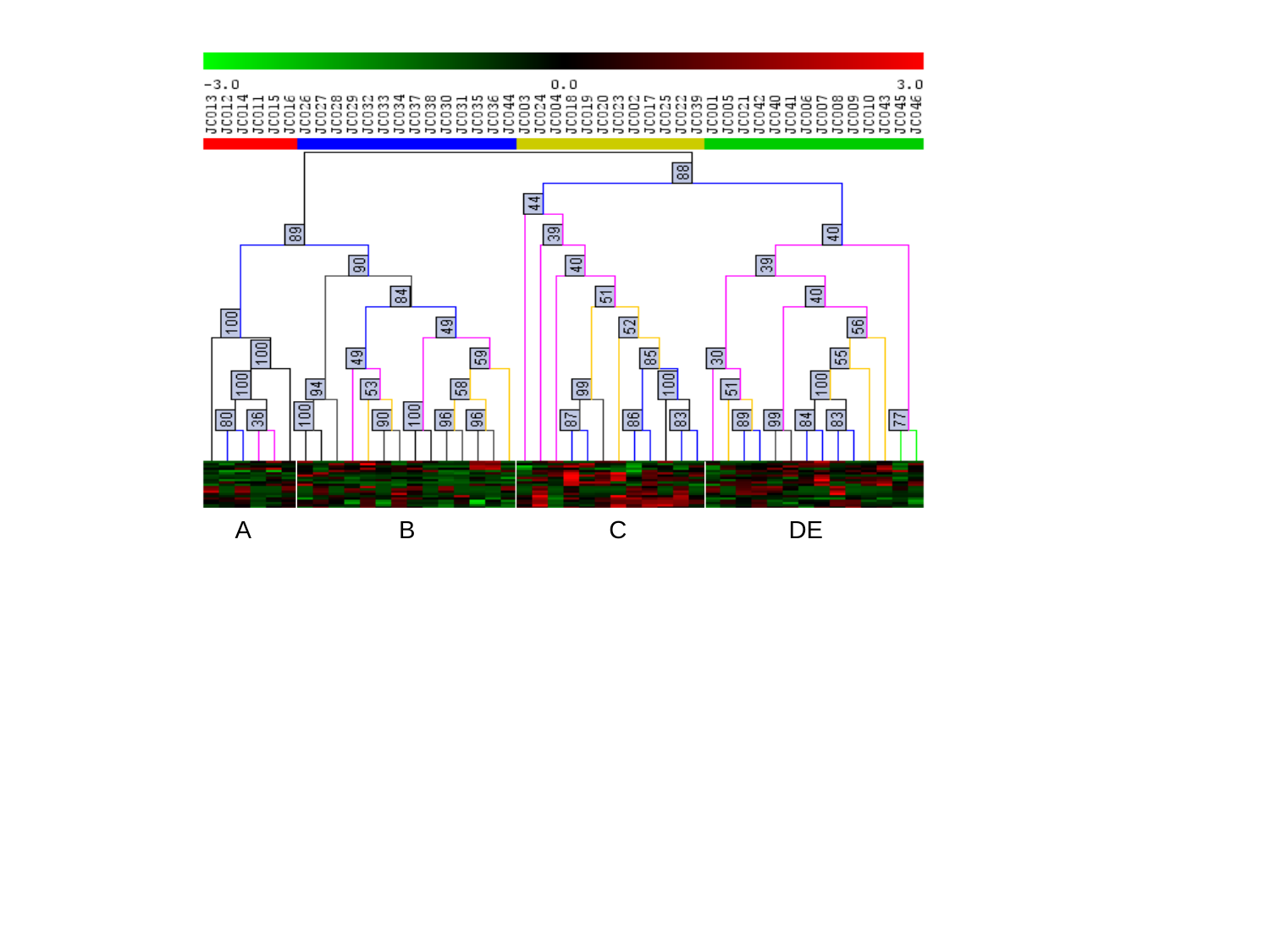

A
B
C
DE

Supplement: Figure S5 — Bootstrap analysis. Bootstrap analysis of unsupervised two-way hierarchical cluster analysis of MB46 data series of Thompson et al. [30] using the 1500 most differentially expressed genes shows that clusters A, B, C, and D/E are indeed distinct clusters. Bootstrap support is 88–100% that the 4 clusters are different from each other. (0.43 MB PPT) [file pone.0003088.s005.ppt]

## Slide 1
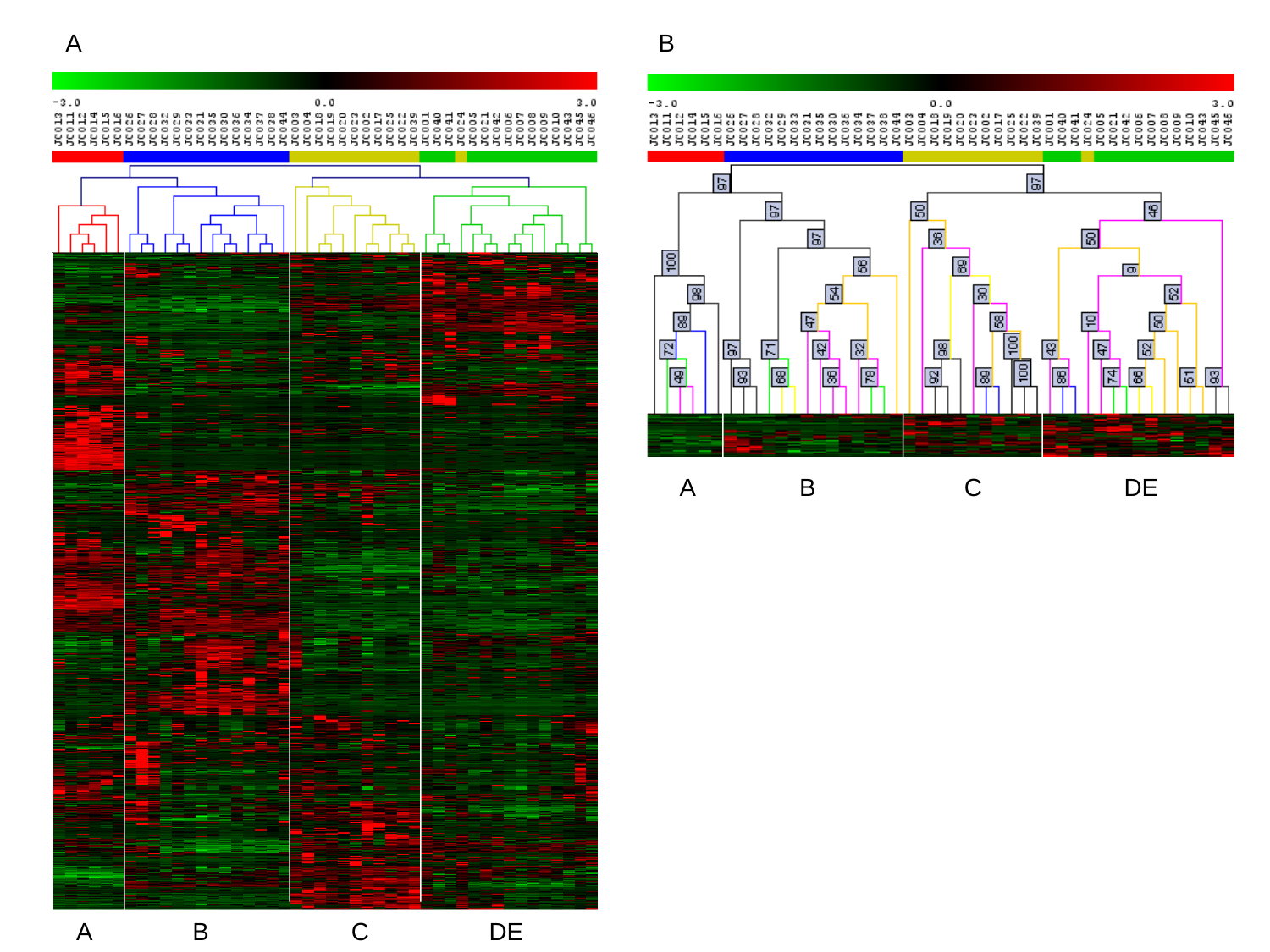

A
B
A
B
C
DE
A
B
C
DE

Supplement: Figure S7 — Cluster analysis. A. Unsupervised two-way hierarchical cluster analysis of 46 medulloblastomas from Thompson data series [30] using the 897 genes present at the HG-U133A GeneChip and present in the 1500 gene dataset selected as most differentially expressed in the MB62 data series. Results show that also with the genes selected from the MB62 data series 4 distinct clusters are identified in the MB46 data series containing the same samples as in Figure 7. Only one sample (JCO24) switched from cluster C to cluster D/E. B. Bootstrap analysis of unsupervised two-way hierarchical cluster analysis of MB46 data series [30] using the 897 genes present at the HG-U133A GeneChip and present in the 1500 gene dataset selected as most differentially expressed in the MB62 data series. The results show that also when these genes are used for clustering the 4 clusters are distinct clusters. (0.33 MB PPT) [file pone.0003088.s007.ppt]
