# Supplementary material for: Integrated Genomics Identifies Five Medulloblastoma Subtypes with Distinct Genetic Profiles, Pathway Signatures and Clinicopathological Features
Source: PLoS One. 2008 Aug 28;3(8):e3088. doi: 10.1371/journal.pone.0003088 (PMC2518524; doi:10.1371/journal.pone.0003088)
Supplement: Figure S2 — PAGE analysis for clusters D and E in MB62 dataseries shows higher levels of expression for cell cycle genes and other proliferation related genes in cluster E. Red dots in the graphs show the average expression for a gene in each cluster. The grey clouds around each dot indicate the standard deviation of expression. (0.27 MB PPT) [file pone.0003088.s002.ppt]

## Slide 1
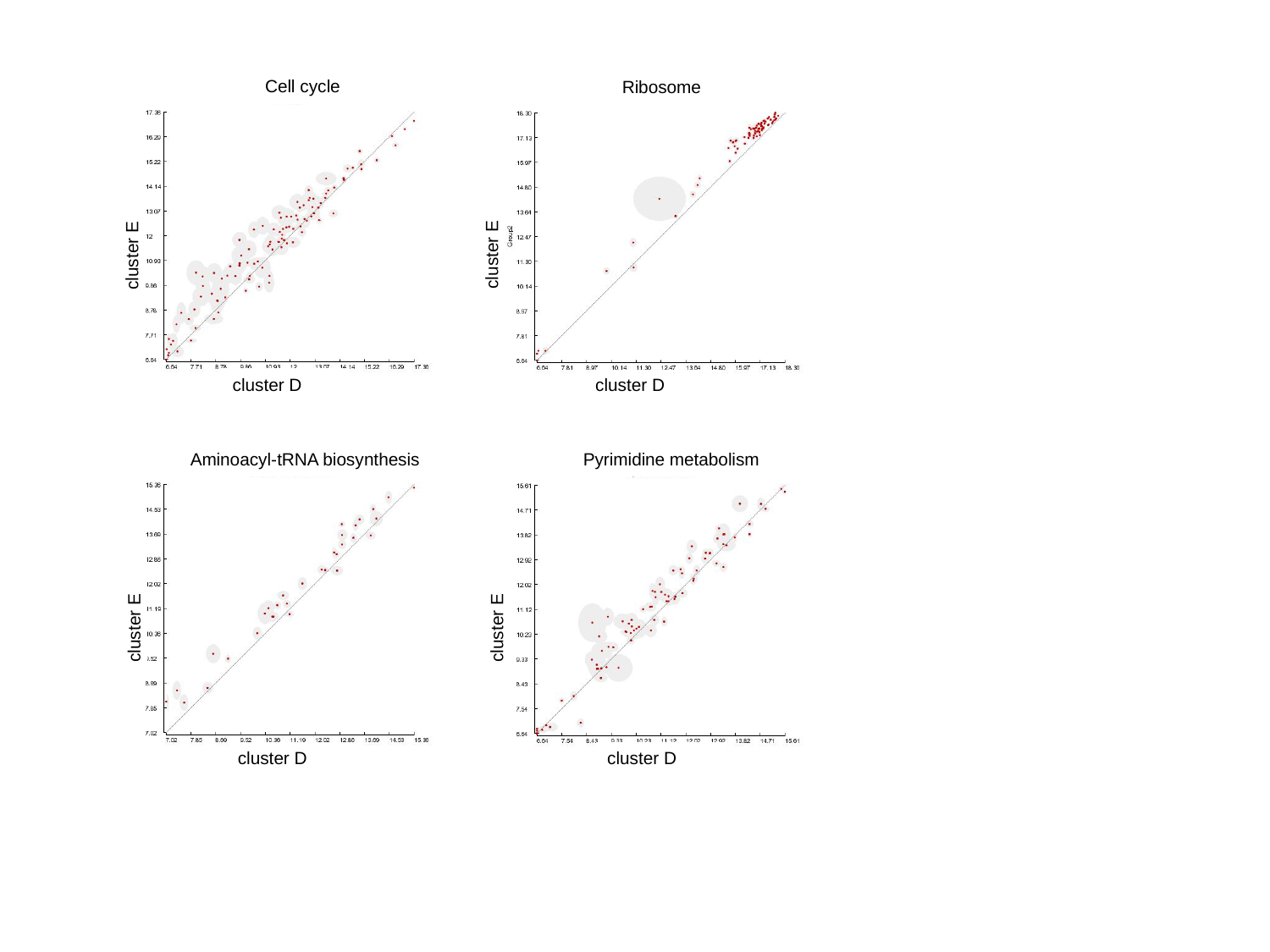

Cell cycle
Ribosome
cluster E
cluster E
cluster D
cluster D
Aminoacyl-tRNA biosynthesis
Pyrimidine metabolism
cluster E
cluster E
cluster D
cluster D
