# Supplementary material for: Integrated Genomics Identifies Five Medulloblastoma Subtypes with Distinct Genetic Profiles, Pathway Signatures and Clinicopathological Features
Source: PLoS One. 2008 Aug 28;3(8):e3088. doi: 10.1371/journal.pone.0003088 (PMC2518524; doi:10.1371/journal.pone.0003088)
Supplement: Figure S6 — Expression data of same selected marker genes from Figure 3 in molecular subtypes of MB46 data series of Thompson et al. [30] show that the subtypes identified in the MB46 data series represent the same subtypes as identified in the MB62 data series. Expression data (vertical axes) are shown for each tumor (indicated with colored circles) for each medulloblastoma subtype. A. Type A markers; B. Type B markers; C. In 2C markers are shown that are expressed either in subtype A and B together or in subtype C, D, and E together; D. Type C and/or CD markers; E. Type DE markers; F. Type E markers. Expression of RUNX2, OTX2, LEMD1, and ZNF179 cannot be shown for the MB46 data series, since the HG-U133A GeneChip does not contain probe sets for these genes. (0.44 MB PPT) [file pone.0003088.s006.ppt]

## Slide 1
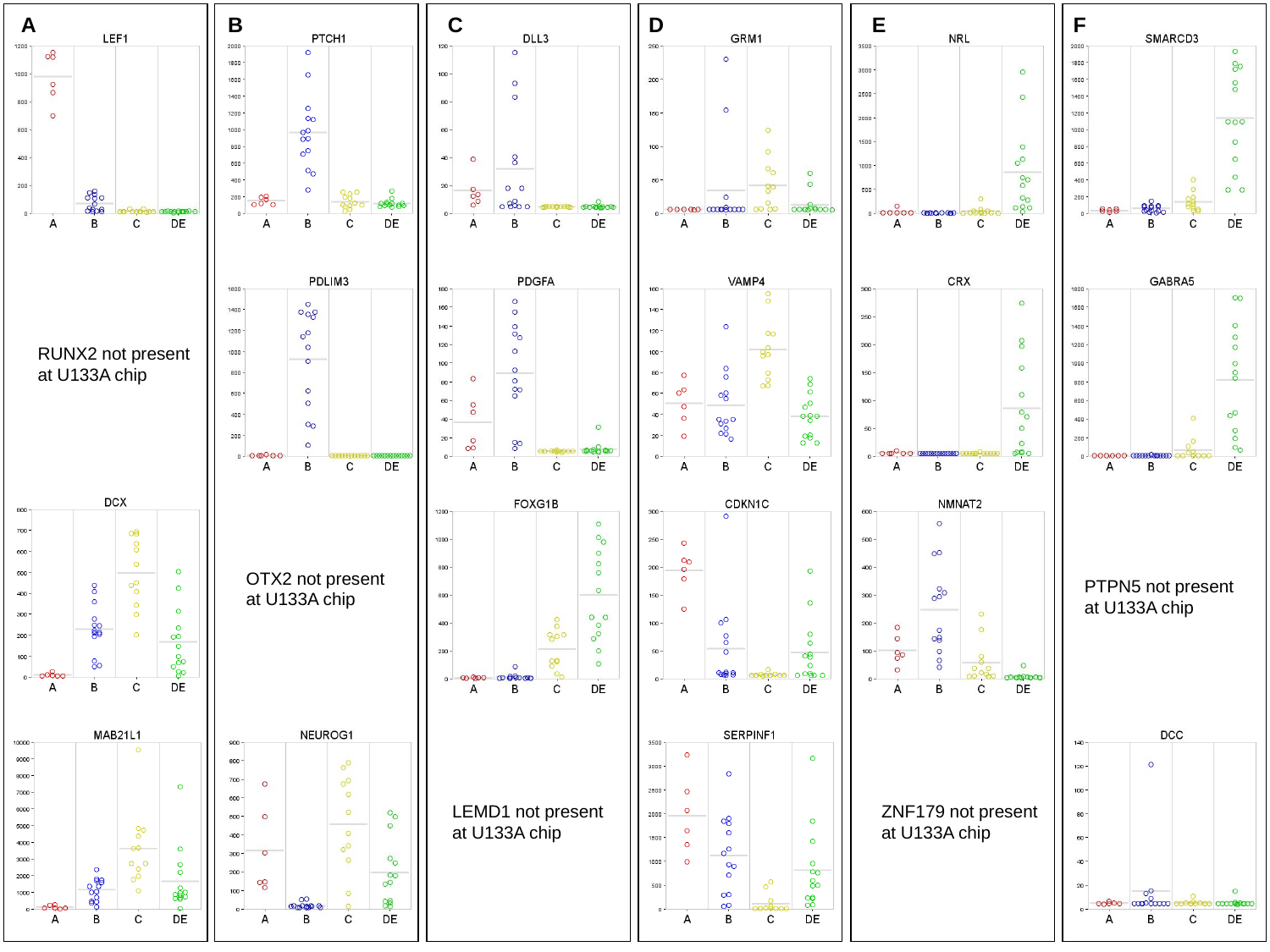

A
B
C
D
E
F
RUNX2 not present
at U133A chip
OTX2 not present
at U133A chip
PTPN5 not present
at U133A chip
LEMD1 not present
at U133A chip
ZNF179 not present
at U133A chip
